# Activation of GM-CSF and TLR2 signaling synergistically enhances antigen-specific antitumor immunity and modulates the tumor microenvironment

**DOI:** 10.1136/jitc-2021-002758

**Published:** 2021-10-01

**Authors:** Wan-Lun Yan, Chiao-Chieh Wu, Kuan-Yin Shen, Shih-Jen Liu

**Affiliations:** 1 Graduate Institute of Life Sciences, National Defense Medical Center, Taipei, Taiwan; 2 National Institute of Infectious Diseases and Vaccinology, National Health Research Institutes, Miaoli County, Taiwan; 3 School of Dentistry, Tri-Service General Hospital and National Defense Medical Center, Taipei, Taiwan; 4 Graduate Institute of Biomedical Sciences, China Medical University, Taichung, Taiwan; 5 Graduate Institute of Medicine, Kaohsiung Medical University, Kaohsiung, Taiwan

**Keywords:** adjuvants, immunologic, tumor microenvironment, vaccination

## Abstract

**Background:**

The major challenge of antitumor immunotherapy is dealing with the immunosuppressive tumor microenvironment, which involves immature myeloid cell accumulation that results in T cell dysfunction. Myeloid cell activation is induced by Toll-like receptor agonists. Additionally, granulocyte/macrophage colony stimulating factor (GM-CSF) promotes myelopoiesis and recruits myeloid cells. Here, we combined the Toll-like receptor 2 (TLR2) agonist lipoprotein and GM-CSF to assess whether this bifunctional immunotherapy has synergistic effects on myeloid cells and could be further developed as a therapeutic intervention that enhances the antitumor response.

**Methods:**

We investigated the synergistic effects of biadjuvanted tumor antigen on antigen-presenting cell (APC) activation in bone marrow-derived dendritic cells. Furthermore, therapeutic efficacy was monitored in different tumor models treated via intratumoral or subcutaneous administration routes. The immune effects of the bifunctional fusion protein on myeloid cells in the tumor mass and draining lymph nodes were analyzed by flow cytometry. The induction of cytotoxic T lymphocytes was evaluated via intracellular cytokine levels, perforin/granzyme B staining and an in vivo killing assay.

**Results:**

The TLR2 agonist lipoprotein combined with GM-CSF synergistically induced DC maturation, which subsequently enhanced antitumor immunity. In addition, rlipoE7m-MoGM modulated tumor-infiltrating myeloid cell populations. Vaccination with rlipoE7m-MoGM therapy increased the number of CCR7^+^CD103^+^ cDC1s, whereas the number of suppressive tumor-associated macrophages was reduced in the tumor lesions. Consistent with this observation, proliferating antigen-specific CD8^+^ T cells are highly infiltrated within the tumor, and the expression of IFN-r and perforin was most pronounced within antigen-specific CD8^+^ T cells in mice administered rlipoE7m-MoGM therapy. This finding corresponded with observation that the combination of a TLR2 agonist and GM-CSF provides increased antitumor activity by inhibiting established tumor outgrowth and protecting against metastatic cancer compared with a TLR2 agonist alone. Importantly, tumor growth inhibition was not due to the direct effects of the TLR2 agonist or GM-CSF but was instead due to the induction of antigen-specific immunity.

**Conclusions:**

The combination of a TLR2 agonist and GM-CSF has synergistic effects that inhibit tumor growth and modulate tumor-infiltrating APCs. This therapeutic approach could be applied to other tumor antigens to treat different cancers.

## Background

Recent revolutionized success in cancer immunotherapy is based on major advances in checkpoint blockade therapy.^1^ However, tumor escape from the immune system remains a major barrier to mounting an effective antitumor response.^2^ This adaptive immune resistance results from failure of intrinsic tumor suppressor mechanisms in which recruitment of immature myeloid cells, secretion of immunosuppressive cytokines, such as interleukin 10 (IL-10), transforming growth factor beta (TGF-β) and vascular endothelial growth factor, and expression of immune checkpoint receptors on T cells constitute an immunosuppressive tumor microenvironment (TME) to suppress cytotoxic T lymphocyte (CTL) function. Given that the capacity of tumor cells to create an immunosuppressive milieu by accumulating immature myeloid cells provides inadequate T cell stimulation and exerts a tolerogenic T cell response, the cooperation of activated myeloid cells and T cells in tumors is critical to optimize the antitumor response. Dendritic cells (DCs) are the most predominant antigen-presenting cells of myeloid populations and have the ability to capture, process and cross-present exogenous antigens, which subsequently induce antitumor responses. Notably, in tumors, DC activation is essential for CTL recruitment and function even though DCs are a rare population, indicating that activation of DCs is essential to increasing the prevalence of tumor-specific T cells in the TME.^3^ Hence, modulating immunosuppressive myeloid cells to reinvigorate the TME into an immunogenic milieu may be a useful strategy for breaking T cell tolerance.

Various therapeutic strategies have been devised to activate myeloid cells. For instance, certain Toll-like receptor (TLR) ligands have been applied to activate myeloid cells appropriately to induce a robust antitumor response.^4^ This activation has been observed after direct targeting of DCs through bacterial lipoprotein, a specific TLR2 agonist. Previously, we developed recombinant lipoprotein (rlipo) technology with a mutated HPV16 E7 tumor antigen (rlipoE7m) that can activate DCs in vitro and in vivo through TLR2 signaling and eradicate early stage TC-1 tumor progression in mice.^5^ However, tumor escape commonly occurs after priming TLR2 agonist therapy due to immature myeloid cell accumulation.^6^ Thus, the most direct method to disrupt the immunosuppressive tumor milieu involves combining cytokines that can recruit and promote DC development. To this end, we propose another strategy to amplify the effects of TLR2 agonists on T cells.

Given its extensive therapeutic history and good safety, granulocyte/macrophage colony stimulating factor (GM-CSF) is a potent immunostimulatory cytokine that recruits and mobilizes myeloid cells and promotes myelopoiesis because the GM-CSF receptor (GM-CSFR) is mainly expressed by myeloid cells.^7 8^ Although GM-CSF is dispensable for homeostatic myelopoiesis in the steady state, upregulation of GM-CSF activates DCs and macrophages, which polarizes these cells into a proinflammatory phenotype during acute inflammation^9^ and governs the development of the T helper-1 (T_H_-1)-type response.^10^ In addition, previous results suggest that GM-CSF is essential for CD103^+^ conventional type 1 DC (cDC1) development and tumor antigen cross-presentation, which subsequently induce T_H_-1 effector cell responses.^11^ While the pleiotropic activity of GM-CSF has also shown contrasting clinical benefits in patients with cancer, aberrant secretion of GM-CSF promotes tumor progression by driving a chronic inflammatory response. These findings point to combinatorial adjuvants that simultaneously recruit and activate DCs required to break CD8^+^ T cell tolerance in the TME.

In the current study, we developed a bifunctional fusion protein rlipoE7m-MoGM in an attempt to further improve antitumor efficacy and TME modulation. We leveraged GM-CSF to mature and recruit DCs in the tumor and simultaneously activated TLR2 on these cells. Our results revealed that TLR2 agonists and GM-CSF cooperated to activate and recruit DCs, which substantially induced a therapeutic antitumor response in an established TC-1 tumor model. Here, rlipoE7m-MoGM showed better antitumor effects than rlipoE7m alone. The possible effector mechanisms of the bifunctional fusion proteins are discussed.

## Methods

### Mice

Female C57BL/6 mice were purchased from the National Laboratory Animal Center (National Applied Research Laboratories, Taiwan). All mice were maintained under speciﬁc pathogen-free conditions at the Laboratory Animal Center of the National Health Research Institutes and used at 6–8 weeks of age in this study.

### Cell line

The TC-1 cell line (lung epithelial cells from C57BL/6 mice transformed with HPV16 E6/E7 and the c-Ha-ras oncogene) was kindly provided by Dr T-C Wu (John Hopkins University, USA). TC-1 cells were maintained in RPMI-1640 medium (HyClone) supplemented with 10% (v/v) heat-inactivated fetal bovine serum (HyClone), 50 units/mL penicillin/streptomycin, 1 mM sodium pyruvate, 25 mM 4-(2-hydroxyethyl)-1-piperazineethanesulfonic acid (HEPES, Biological Industries) and 55 µM β-mercaptoethanol. E.G7-OVA (ATCC, CRL-2113) is a derivative of mouse thymoma EL4 cells transfected with the ovalbumin (OVA) plasmid. The E.G7-OVA cells were cultured in RPMI-1640 medium supplemented with 10% (v/v) heat-inactivated fetal bovine serum, 50 units/mL penicillin/streptomycin, 1 mM sodium pyruvate, 20 mM HEPES, 50 µM β-mercaptoethanol and 0.4 mg/mL G418. B16F10-OVA melanoma cells were cultured in Dulbecco's Modified Eagle Medium (DMEM, Gibco) supplemented with 10% (v/v) heat-inactivated fetal bovine serum, 50 units/mL penicillin/streptomycin and 1 mg/mL G418. All cells were maintained at 37°C in 5% CO_2_ and determined to be mycoplasma negative before the animal study.

### Production and puriﬁcation of rlipoE7m-MoGM

The DNA sequence encoding the mouse GM-CSF protein Ala18-Lys141 (NP_034099.2) was codon optimized for expression in *Escherichia coli*. The MoGM coding region was amplified by PCR using gene synthesis of wild-type (WT) MoGM (GeneScript) as a template with primers as follows: forward 5’-CTCGAGGCGCCGACCCGTAG-3’; reverse 5’-CTCGAGTTTCTGGCCCGG TTTTTT-3’ (Xho I site, underlined). The mutated human papillomavirus type 16 E7 (E7m) gene with a lapidated sequence was obtained as previously described.^5^ To create the rlipoE7m-MoGM expression plasmids, amplified cDNA was subcloned into the pET-22B(+) vector (Novagen) to generate a 16E7m construct with a lipid moiety at the N-terminus, MoGM and an additional hexahistidine tag (HisTag) at the C-terminus (rlipoE7m-MoGM). To express the recombinant protein, the *Escherichia coli* strain C43(DE3) (Lucigen) was transformed with the expression plasmid and cultured at 37°C in LB broth until an OD_600_ of 0.6 was reached. Then, a 50-fold dilution of bacterial suspension was subcultured into M9 medium and incubated at 37°C before induction. The expression of rlipoE7m-MoGM was induced with 1 mM isopropyl β-d-1-thiogalactopyranoside for 72 hours, and the cells were harvested by centrifugation. The harvested cells were disrupted in a French press (Constant Systems, Daventry, UK) at 27 Kpsi in homogenization buffer (20 mM Tris (pH 8.0), 50 mM sucrose, 500 mM NaCl and 10% glycerol). The cell lysate was collected by ultracentrifugation (32,000 rpm, 40 min). The inclusion bodies containing rlipoE7m-MoGM were dissolved in 6 M guanidine HCl (GuHCl) extraction buffer (6 M GuHCl, 50 mM Tris (pH 8.9), 300 mM NaCl and 1% Triton X-100). The extracted supernatant was loaded onto a Ni-NTA resin column (Qiagen). rlipoE7m-MoGM was eluted with phosphate-buffered saline (PBS) containing 500 mM imidazole and then dialyzed against 50 mM ammonium bicarbonate. The endotoxin levels of puriﬁed rlipoE7m-MoGM were determined using the Limulus amebocyte lysate assay (Associates of Cape Cod), and all measured endotoxin levels were less than 3.9 EU/mg.

### Bone marrow-derived dendritic cell (BMDC) culture and stimulation

Briefly, bone marrow cells were flushed from the femurs and tibias of C57BL/6 mice and cultured in complete media (RPMI-1640 media supplemented with 10% (v/v) heat-inactivated fetal bovine serum, 50 units/mL penicillin/streptomycin, 1 mM sodium pyruvate, 25 mM HEPES and 55 µM β-mercaptoethanol) including 20 ng/mL recombinant GM-CSF (PeproTech) as previously described.^12^ New complete medium supplemented with GM-CSF was added on day 3. Then, on day 6, the floating cells in the culture supernatant and loosely adherent cells harvested by gentle washing with PBS were collected and treated with recombinant proteins for 16 hours. The expression of CD80, CD86 and CCR7 on BMDCs (pregated as CD11c^+^MHCII^+^) was analyzed by flow cytometry, and the fold change in geometric means relative to that of the medium control was calculated. BMDC culture supernatants were collected, and the concentrations of the cytokines IL-1β, IL-6, IL-12p70 and tumor necrosis factor alpha (TNF-α) were determined by ELISA.

### Tumor model

C57BL/6 mice were subcutaneously (s.c.) inoculated with 2×10^5^ TC-1 cells in 100 µL PBS in the right flank. When the tumor size reached 50–100 mm^3^ (approximately 14 days), recombinant proteins (1 or 4 nmol) were intratumorally injected on days 14, 16 and 18. Alternatively, recombinant proteins (1 or 4 nmol) were s.c. injected over the shoulder of mice. Mice were s.c. implanted with 2×10^5^ E.G7 tumor cells in the flank and s.c. immunization on day 7 post-tumor challenge. Tumors were monitored every 2–3 days, and tumor volume was calculated using the following formula: (length×width^2^/2). B16F10-OVA (5×10^5^) melanoma cells were intravenously injected into mice, and the cells were allowed to develop nodules in the lungs for 19 days. The number of metastatic nodules was determined using particle analysis in the ImageJ software.

### CD8^+^ T cell depletion

In vivo CD8^+^ T cell depletion was performed using a purified anti-CD8a (53–6.7) blocking antibody purchased from Biolegend. TC-1 tumor-bearing mice were depleted of CD8^+^ cells using 0.5 mg of anti-CD8a or control rat IgG antibodies injected intraperitoneally 1 day prior to s.c. therapeutic immunization on day 14.

### Cell stimulation and flow cytometry analysis of tumor-infiltrating cells and TdLNs

Tumor tissues and tumor-draining lymph nodes (TdLNs) were minced and enzymatically digested with 200 U type IV collagenase (Gibco) at 37°C for 30 min to obtain single-cell suspensions. Cell viability was assessed by staining with ﬁxable Live/Dead Zombie Yellow (BioLegend) in PBS. For RAH-specific T cell staining, single-cell suspensions were stained with a PE-labeled RAH tetramer (D^b^-RAHYNIVTF, Beckman Coulter) for 15 min in staining buffer (2% FBS and 0.05% sodium azide in PBS) at room temperature. For surface marker staining, Fc receptors were blocked with αCD16/CD32 (2.4G2) for 10 min and stained with the indicated antibodies for 30 min in staining buffer at 4°C. The following antimouse antibodies were purchased from BD Pharmingen, eBioscience or BioLegend: αCCR7 (4B12), αCD103 (2E7), αCD11b (M1/70), αCD11c (N418), αCD19 (1D3), αCD4 (Gk1.5), αCD45 (30-F11), αCD64 (X54-5/7.1), αCD8a (53–6.7), αGranzyme B (NGZB), αI-A/I-E (M5/114.152), αIFN-γ (XMG1.2), αIL-6 (MP5-20F3), αIL-10 (JES5-16E3), αKi67 (16A8), αLy6C (HK1.4), αLy6G (1A8), αPerforin (S16009B) and αTGF-β (TW7-16B4). For T cell stimulation assays, tumor cells were incubated in the presence of E7_49-57_ peptides (RAHYNIVTF, 10 μg/mL), phorbol 12-myristate 13-acetate (PMA) and ionomycin with Golgi inhibitors (brefeldin A, BioLegend) for 4–6 hours. For evaluation of myeloid cell function, tumor cells were stimulated with lipopolysaccharide (LPS; 1 mg/mL) for 8 hours in the presence of Golgi inhibitors. Unstimulated cells were used as negative controls. All ﬂow cytometry experiments were performed on an Attune NxT ﬂow cytometer (Thermo Fisher), and ﬂow cytometry data were analyzed with FlowJo (TreeStar).

### In vivo killing assay

Splenocytes from C57BL/6 mice were isolated and pulsed with RAH peptides (RAHYNIVTF) or irrelevant EBV LMP2 peptides (SSCSSCPLSK) at 37°C for 30 min to produce target cells. Then, the RAH-pulsed target cells and irrelevant peptide-pulsed control cells were labeled with 10 µmol/L and 1 µmol/L CellTrace CFSE (Thermo), respectively. After washing with PBS twice, the target cells and control cells were mixed at a 1:1 ratio in PBS and injected intravenously into immunized mice. After 16 hours, splenocytes were isolated and analyzed by flow cytometry. Antigen-speciﬁc killing was calculated using the following equation: 100-(100×(carboxyfluorescein succinimidyl ester (CFSE)^hi^/CFSE^lo^)/(nonimmunized mice CFSE^hi^/CFSE^lo^)).

### Statistical analysis

All statistical analyses were performed with Prism 6 software (GraphPad). For continuous tumor volume observations, statistical signiﬁcance was calculated by two-way analysis of variance (ANOVA) with Tukey’s correction for multiple comparisons. For multiple group comparisons, analyses of the mean and SE of the mean (SEM) values were calculated using one-way ANOVA for multiple comparisons. ^∗^P<0.05, ^∗∗^p<0.01, and ^∗∗∗^p<0.001 were considered statistically significant.

## Results

### Production and functional identification of rlipoE7m-MoGM

**Figure 1:**
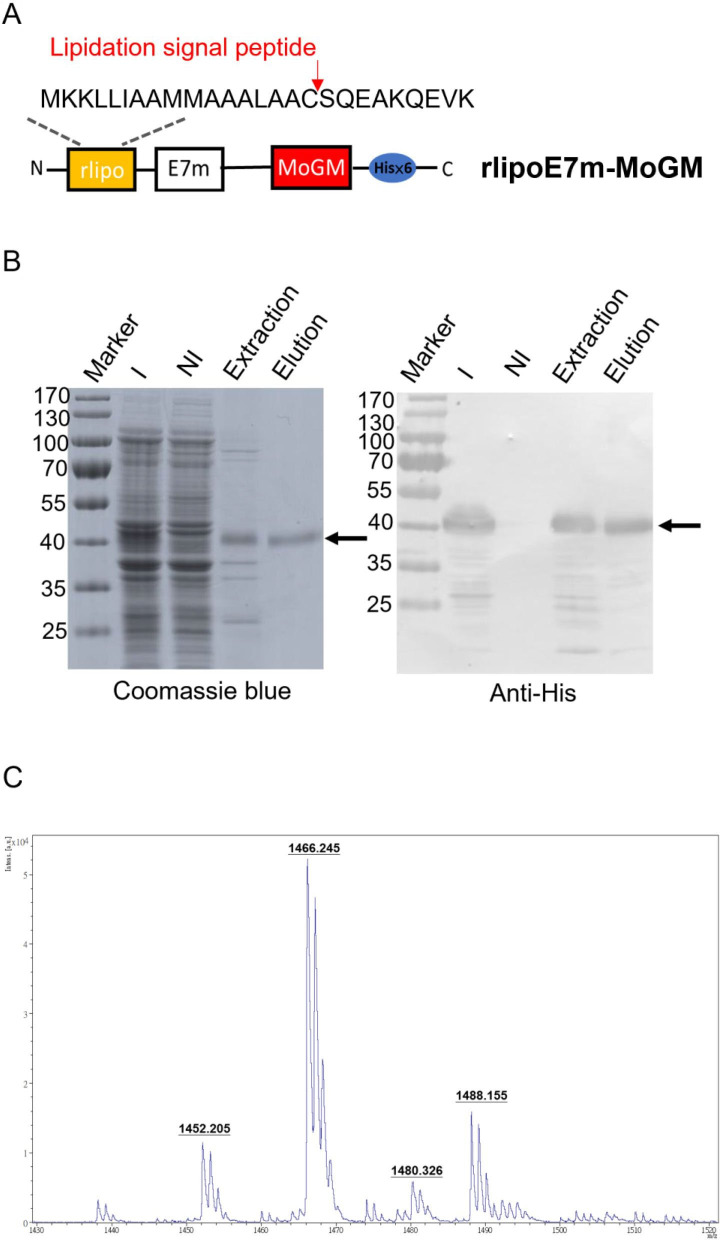
Design, production and characterization of rlipoE7m-MoGM. (A) Schematic diagram of rlipoE7m-MoGM. A mutated human papillomavirus type 16 E7 (E7m) sequence was cloned into the pET22b vector along with a lipidation signal peptide at the N-terminus and a hexahistidine tag at the C-terminus with mouse GM-CSF (MoGM). (B) rlipoE7m was expressed in *Escherichia coli* and purified with Ni-NTA. The protein purity at each step was analyzed by 10% SDS-PAGE (left) with detection by anti-His Tag antibodies (right). (C) The N-terminal lipid moiety of rlipoE7m-MoGM was analyzed on a MALDI micro MX mass spectrometer. The MALDI-TOF MS spectra showed three lipid peptide signal peaks: 1452, 1466 and 1480 m*/z.* GM-CSF; granulocyte/macrophage colony stimulating factor; I, IPTG induction; MALDI-TOF; matrix-assisted laser desorption/ionization-time of flight; NI, no induction; SDS-PAGE, sodium dodecyl sulfate-polyacrylamide gel electrophoresis.

To generate recombinant proteins with built-in TLR2 agonists and GM-CSF dual-function activity, we constructed tumor antigens with an N-terminal lipidation signal and fused them to MoGM and a hexahistidine tag at the C-terminus (figure 1A). The recombinant protein was expressed in an *Escherichia coli*-based expression system. Using immobilized metal affinity chromatography, purified rlipoE7m-MoGM was analyzed by 10% sodium dodecyl sulfate-polyacrylamide gel electrophoresis followed by staining with Coomassie blue or immunoblotting with anti-His Tag antibodies (figure 1B). To further determine whether the purified recombinant TLR2 agonist and GM-CSF-containing fusion protein were biologically active, we incubated NFS-60 cells that proliferate in response to GM-CSF with various concentrations of recombinant proteins in vitro. The results showed that both rlipoE7m-MoGM and rlipo-ovalbumin (OVA)-MoGM maintained GM-CSF bioactivity (online supplemental figure S1). In addition, the lipid moiety was identified by matrix-assisted laser desorption/ionization-time of flight mass spectrometry to analyze peptide mass fingerprinting. The results demonstrated three major m/z values of 1452, 1466 and 1480 (figure 1C), representing the lipidated CSQEAK sequence, which was consistent with our previous studies.^13 14^ These results suggest that the purified recombinant protein rlipoE7m-MoGM contained a lipidated moiety and exhibited GM-CSF activity.

10.1136/jitc-2021-002758.supp1 Supplementary data

### rlipoE7m-MoGM synergistically activates BMDCs through TLR2 and GM-CSF signaling

**Figure 2:**
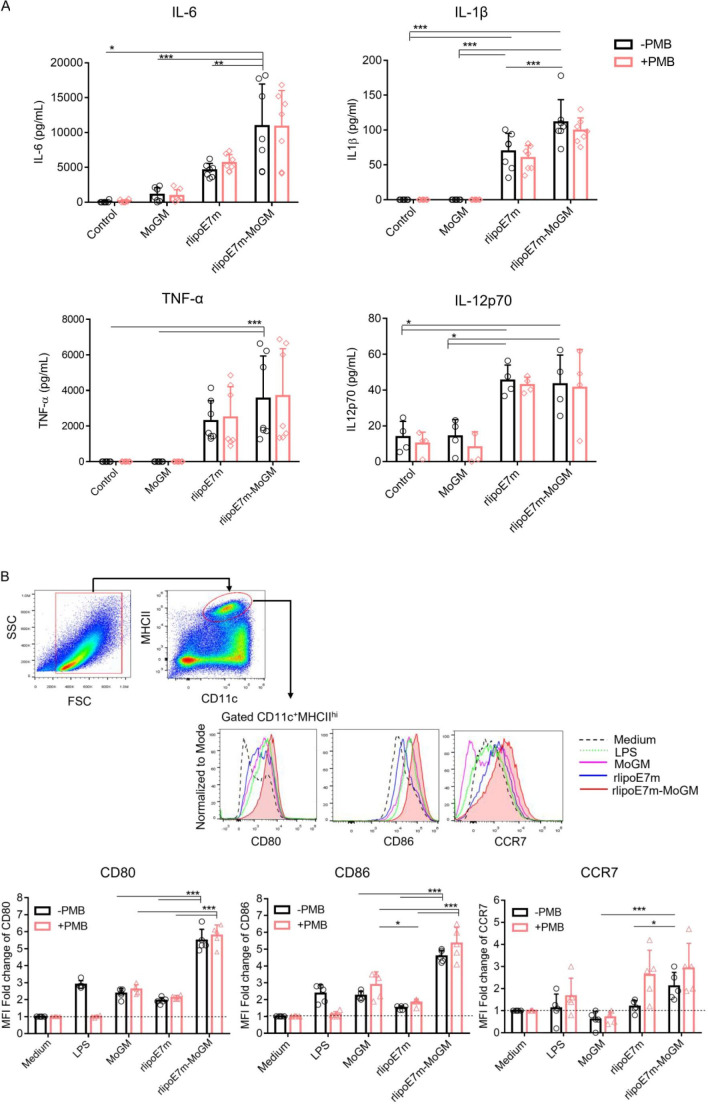
GM-CSF-fused rlipoE7m enhances BMDC activation. (A) BMDCs derived from C57BL/6 mice were stimulated with medium alone and 10 nM recombinant proteins with or without PMB (30 µg/mL) as indicated. After 20 hours of incubation, the culture supernatants of stimulated BMDCs were collected and assayed for IL-6, IL-1β, TNF-α and IL-12p70 by ELISA. (B) BMDCs were collected and gated on CD11c^+^MHCII^hi^ cells to analyze the expression of the surface markers CD80, CD86 and CCR7 by flow cytometry. The gating strategy and one representative experiment are shown. The MFI fold change was defined with the medium alone treatment set as one. The data shown are the mean±SD of n=5 mice and pooled from three independent experiments. *P<0.05 and ***p<0.001 (two-way ANOVA with Tukey correction). ANOVA, analysis of variance; BMDC, bone marrow-derived dendritic cell; GM-CSF, granulocyte/macrophage colony stimulating factor; IL, interleukin; LPS, lipopolysaccharide; MFI, mean fluorescence intensity; OVA, ovalbumin; PMB, polymyxin B.

We next evaluated whether TLR2 agonist and GM-CSF-containing fusion proteins activate BMDCs in a synergistic manner. To this end, BMDCs were stimulated with MoGM, rlipoE7m or rlipoE7m-MoGM for 18 hours. The results showed that rlipoE7m-MoGM-treated WT mouse-derived BMDCs showed increases in the production of the proinflammatory cytokines IL-6 and IL-1β, although TNF-α and IL-12p70 production was not pronounced compared with rlipoE7m-treated BMDCs (figure 2A). To determine whether a TLR2 agonist and GM-CSF cooperate to stimulate BMDC activation, we validated the expression of costimulatory molecules and the migratory chemokine CC-chemokine receptor 7 (CCR7). The results showed that WT BMDCs treated with rlipoE7m-MoGM exhibited a fivefold to twofold increase in the expression of the costimulatory molecules CD80/CD86 and migratory marker CCR7, respectively, but rlipoE7m alone did not produce the same effect (figure 2B). To further exclude any minimal endotoxin effect on BMDC proinflammatory cytokine production and activation, polymyxin B (PMB) was mixed with the recombinant proteins to stimulate BMDCs. The results showed an increase in the production of the proinflammatory cytokines IL-6 and IL-1β and the expression of CD80/CD86 and the migratory marker CCR7 in BMDCs that were similar regardless of the presence or absence of PMB, indicating that TLR2 agonists and GM-CSF cooperate to activate DCs (figure 2A, B). Together, these data suggest that activation of TLR2 signaling is mediated through the lipid moiety of the lipoprotein and that TLR2 agonists and GM-CSF work in concert to induce downstream proinflammatory cytokine/chemokine expression, whereas TLR2 agonists alone have limited effects on BMDC activation.

### Intratumoral rlipoE7m-MoGM therapy induces therapeutic antitumor immunity against established tumors at the treated site and untreated distant sites

**Figure 3:**
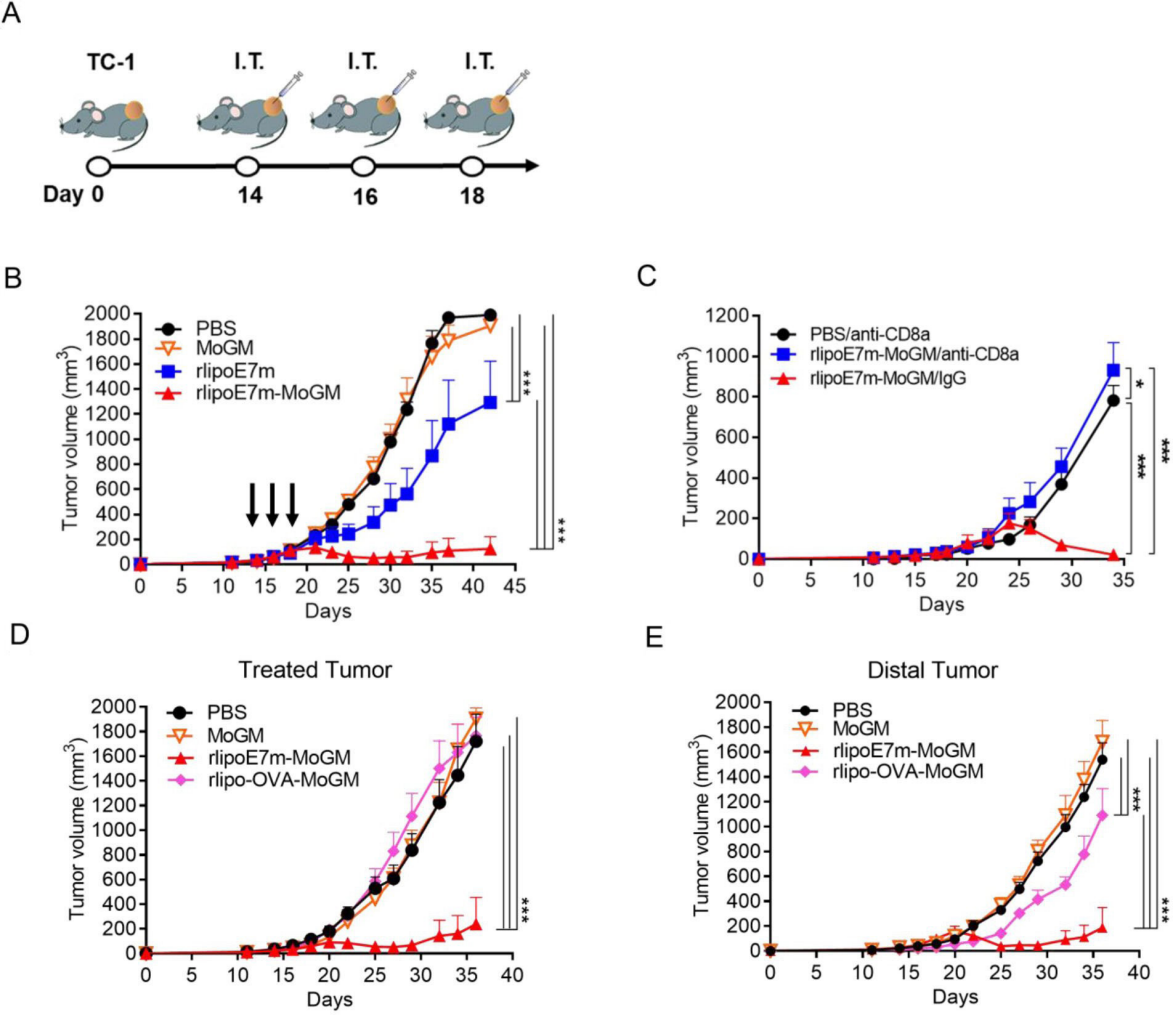
Intratumoral injection of rlipoE7m-MoGM induces both local and systemic antigen-specific antitumor responses. (A) The experimental design is shown. (B) TC-1 tumor-bearing mice were i.t. treated with multiple injections of 1 nmol recombinant proteins on days 14, 16 and 18. Tumor size was monitored every 2–3 days. (C) TC-1 tumor-bearing mice were i.p. injected with 0.5 mg of anti-CD8a or rat IgG one day before immunization. (D) Mice were s.c. inoculated TC-1 tumor cells (2×10^5^ /mouse) concurrently into both sides of the flank. PBS or the GM-CSF fusion protein rlipo-OVA-MoGM or rlipoE7m-MoGM (1 nmol) was injected into the left flank on days 14, 16 and 18. Tumor size was monitored every 2–3 days. (E) The size of the untreated distal tumor in the right flank was monitored. Data are shown as the mean±SEM. n=6–8 mice per group from two independent experiments in (B) and n=5–6 mice per group from a single experiment in (C, D and E). *P<0.05 and ***p<0.001 (two-way ANOVA with Tukey’s correction). ANOVA, analysis of variance; GM-CSF, granulocyte/macrophage colony stimulating factor; i.p., intraperitoneal; i.t., intratumoral; OVA, ovalbumin; s.c., subcutaneous.

Given the capacity of tumor cells to create an immunosuppressive milieu in which immature myeloid cell accumulation provides inadequate activation of CD8^+^ T cells that leads to tumor-specific T cell dysfunction, we evaluated the therapeutic potential of direct intratumoral (i.t.) administration of rlipoE7m-MoGM to late-stage tumors. Therefore, mice were s.c. inoculated with TC-1 tumor cells followed by i.t. injection of recombinant proteins on days 14, 16 and 18; tumor growth was monitored (figure 3A). Remarkably, we found that i.t. rlipoE7m-MoGM therapy resulted in robust synergistic antitumor efficacy with tumor regression 7 days after the final treatment. In total, 2 out of 8 mice were tumor-free, and all animals survived more than 40 days. In contrast, MoGM monotherapy failed to inhibit the growth of established tumors, and rlipoE7m treatment showed a marginal tumor growth delay (figure 3B). Next, we assessed memory immunity, and cured mice treated with rlipoE7m-MoGM were rechallenged with TC-1 tumor cells in the contralateral flank at day 29. No mice exhibited tumor growth at day 21 after tumor rechallenge, whereas naïve mice developed growing tumors (online supplemental figure S2). To further determine whether the rlipoE7m-MoGM-induced antitumor effect is CD8^+^ T cell dependent, TC-1 tumor-bearing mice were depleted of CD8^+^ T cells using an anti-CD8α antibody one day before i.t. immunization. The results showed that CD8^+^ cell depletion facilitated tumor outgrowth in mice receiving i.t. rlipoE7m-MoGM therapy, whereas tumor regression was observed in mice treated with control rat IgG antibody. These findings suggest that antitumor activity induced by i.t. rlipoE7m-MoGM is CD8^+^ T cell driven (figure 3C).

To further explore whether i.t. administration of the TLR2 agonist and GM-CSF-containing fusion proteins would lead to a systemic antitumor response, we used a bilateral tumor approach. We treated one side tumor to determine the impact of therapy on the distant tumor concurrently implanted in the contralateral flank of mice. We found that i.t. rlipoE7m-MoGM therapy, but not MoGM treatment, conferred a stronger antitumor response, resulting in regression at both the treated tumor site (figure 3D) and untreated distal tumor outgrowth (figure 3E). Moreover, this antitumor effect induced by the dual adjuvants TLR2 agonists and GM-CSF is tumor antigen dependent. To distinguish the therapeutic impact in the unmatched tumor model, we expressed and purified the unmatched tumor antigen OVA with a lipid moiety at the N-terminus and fused it with GM-CSF at the C-terminus (rlipo-OVA-MoGM, online supplemental figure S3). The results showed that unmatched tumor antigen rlipo-OVA-MoGM therapy had no impact on TC-1 tumor growth in either treated tumors or distal tumors, indicating that the antitumor efficacy induced by the combination of the TLR2 agonist and GM-CSF was tumor antigen dependent (figure 3D, E). These data suggest that i.t. rlipoE7m-MoGM therapy induces local antitumor immunity and ultimately triggers systemic regression of distal tumors, and the antitumor activity induced by i.t. rlipoE7m-MoGM is CD8^+^ T cell-driven.

### Intratumoral injection of a TLR2 agonist and GM-CSF-containing fusion protein activates CD103^+^ DCs and decreases the frequency of TAMs in tumors

**Figure 4:**
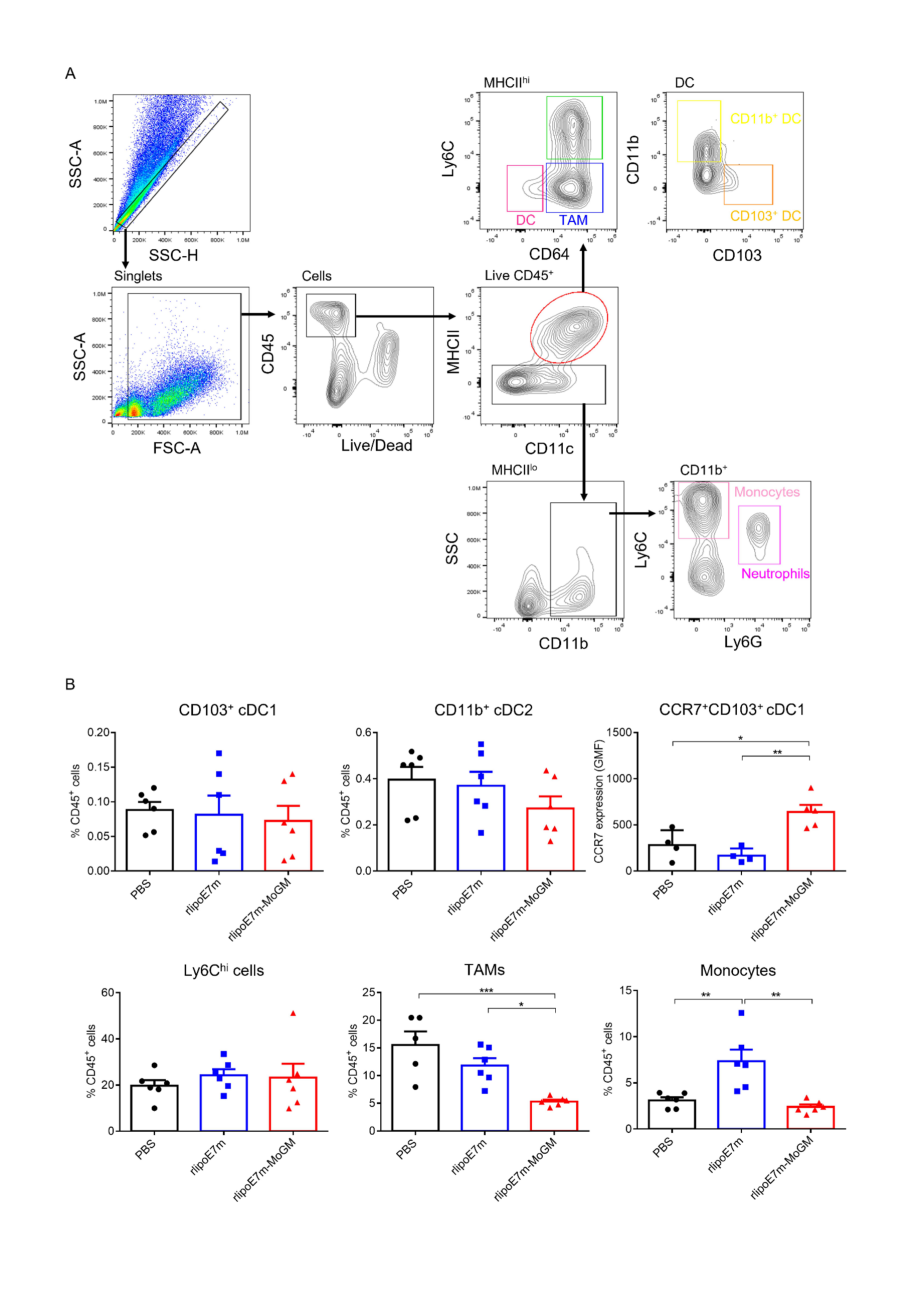
Tumor-infiltrating myeloid cell populations after i.t. treatment with rlipoE7m-MoGM. TC-1 tumor-bearing mice were treated with the indicated recombinant proteins on days 14, 16 and 18. The mice were sacrificed on day 20, and the tumor mass was digested for cell population analysis. (A) The gating strategy of tumor-infiltrating myeloid cells. (B) The frequency of each myeloid cell subset within the CD45^+^ cell population in tumors was analyzed. Data are shown as the mean±SEM of n=4–6 mice per group from two independent experiments. *P<0.05, **p<0.01 and ***p<0.001 (one-way ANOVA with Tukey’s correction). ANOVA, analysis of variance; i.t., intratumoral.

DCs are a heterogeneous population consisting of CD103^+^ or CD8^+^ classical cDC1s, CD11b^+^ classical type 2 DCs (cDC2s), plasmacytoid DCs (pDCs) and monocyte-derived DCs (MoDCs).^15^ Because the tumor milieu prevents i.t. DCs from inducing an effective CD8^+^ T cell response, we further explored the mechanism through which the dual TLR2 agonist and GM-CSF activate the tumor-infiltrating myeloid cell population after i.t. administration. To characterize the in vivo diversity of tumor-infiltrating myeloid cells in response to rlipoE7m-MoGM therapy, we harvested the treated tumors after 2 days of final i.t. treatment. The flow cytometry gating strategy of tumor-infiltrating myeloid cells is shown (figure 4A). We observed that in the tumor lesions, Ly6c^hi^ cells and tumor-associated macrophages (TAMs) were the most abundant infiltrating myeloid cells. Both the CD103^+^ cDC1 and CD11b^+^ cDC2 subsets were found at low frequencies, representing no more than 2% of the CD45^+^ cell population. The tumor lesions of mice receiving rlipoE7m-MoGM therapy showed a reduced number of TAMs, whereas the numbers of CD103^+^ DCs and CD11b^+^ DCs remained unchanged before tumor regression, suggesting that the marked reduction in the TAM population was not an artifact of tumor shrinkage (figure 4B). Although CD103^+^ DCs were not recruited to the tumors, upregulation of CCR7 chemokine receptor expression was observed in CD103^+^ DCs after rlipoE7m-MoGM therapy, suggesting that in vivo i.t. rlipoE7m-MoGM therapy synergistically activates DCs and induces proper DC migration to the TdLNs. We expanded the analysis to TdLNs to clarify the myeloid cell population outside of tumors. However, we found no significant change in each myeloid cell population of TdLNs after treatment (online supplemental figure S4). Herein, we conclude that dual TLR2 agonist and GM-CSF induce local migration of cDC1s and decrease the number of TAMs, which subsequent transform the immunosuppressive TME into a site supporting effective antitumor immunity.

### Administration of dual TLR2 agonist and GM-CSF therapy subcutaneously regresses established tumors in diverse syngeneic cancer models

**Figure 5:**
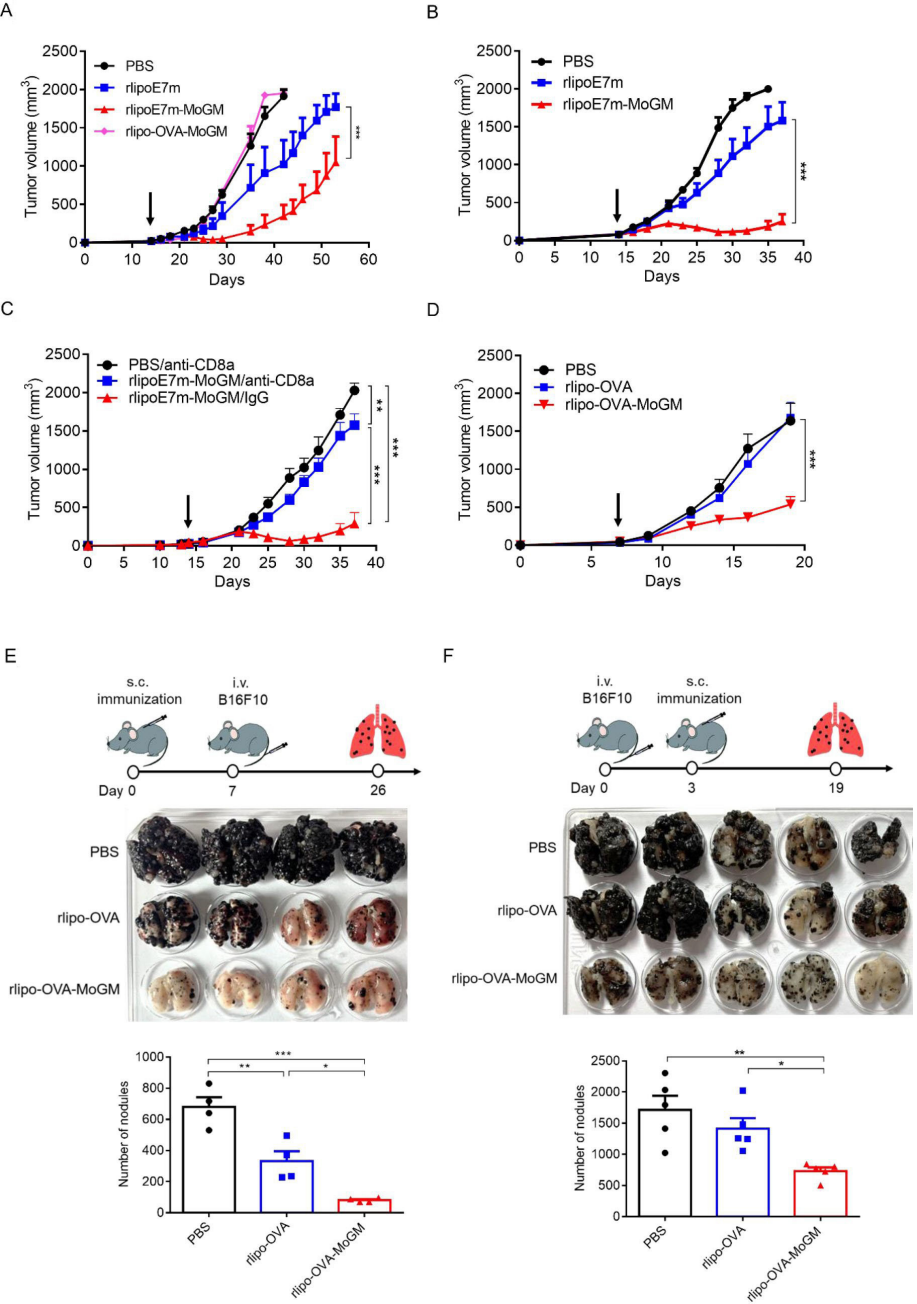
Dual TLR2 and GM-CSF stimulation effectively treated established tumors in diverse syngeneic cancer models. TC-1 tumor-bearing mice (tumor size: 50–100 mm^3^) were s.c. administration once with 1 nmol (A) or 4 nmol (B) indicated recombinant proteins to the backs of mice on day 14 post-tumor challenge. (C) TC-1 tumor-bearing mice were i.p. depleted with anti-CD8a or control rat IgG (0.5 mg) antibodies one day prior to immunization. (D) E.G7 tumor-bearing mice were subject to s.c. administration of 1 nmol of the indicated recombinant proteins to the backs of mice on day 7 post-tumor challenge. Data are presented as the mean±SEM. (E, F) Photos of formalin-fixed lungs showing melanoma metastasis. The number of metastasis tumor nodules in lungs was counted. (E) Mice were s.c. immunized with recombinant proteins, and tumors were challenged with intravenous B16F10-OVA melanoma cells on day 7 postimmunization and analyzed melanoma lung metastasis on day 26 (post-tumor challenge day 19). (F) Mice were administered an intravenous injection of B16F10-OVA melanoma cells on day 0, s.c. received recombinant protein therapy on day 3 and analyzed for melanoma lung metastasis on day 19. n=4–9 mice per group from two independent experiments in (A), n=4–6 mice per group from a single experiment in (B, C, E and F) and from two independent experiments in (D). *P<0.05, **p<0.01 and ***p<0.001 (two-way ANOVA with Tukey’s correction (A–D), one-way ANOVA with Tukey’s correction (E,F)). ANOVA, analysis of variance; GM-CSF, granulocyte/macrophage colony stimulating factor; OVA, ovalbumin; i.p., intraperitoneal; s.c., subcutaneous.

Based on the results showing that direct i.t. immunization with rlipoE7m-MoGM induced significant tumor regression and transformed the immunosuppressive TME, we asked whether systemic administration of rlipoE7m-MoGM induces effective antitumor immunity against established tumors. A previous study showed that systemic rlipoE7m therapy alone induced a moderate antitumor response in a late-stage TC-1 tumor model that expressed the immunodominant E7 antigens.^6^ To test the ability of GM-CSF to enhance the antitumor efficacy of systemic rlipoE7m treatment, TC-1 tumor-bearing mice were s.c. immunized with rlipoE7m-MoGM once on day 14 when the tumor volume reached 50–100 mm^3^. Then, tumor growth was monitored. Following low-dose (1 nmol) rlipoE7m-MoGM therapy, late-stage TC-1 tumors started to regress 10 days after s.c. immunization, but regression did not occur in the PBS control and rlipoE7m alone groups (figure 5A). Additionally, TC-1 tumor-bearing mice immunized with the unmatched tumor antigen rlipo-OVA-MoGM fusion protein failed to develop antitumor responses in a manner indistinguishable from the response of the control tumor-bearing mice treated with PBS (figure 5A). Consistent with i.t. rlipoE7m-MoGM therapy, tumor regression induced by s.c. administration of TLR2 agonists and GM-CSF-containing fusion proteins is antigen dependent. Moreover, a higher dose (4 nmol) of rlipoE7m-MoGM therapy completely inhibited tumor growth (figure 5B). Additionally, the prevalence and potency of cytotoxic effector CD8^+^ T cells was determined in response to rlipoE7m-MoGM immunization. To this end, naïve mice were immunized twice with a 1-week interval, and spleens were collected 7 days after the second immunization. We observed increased IFN-γ production (online supplemental figure S5A) and an increased number of RAH-specific IFN-γ-secreting cells (online supplemental figure S5B). Accordingly, high-dose (4 nmol) rlipoE7m-MoGM immunization induced increased IFN-γ production and reduced IL-5 secretion, suggesting that rlipoE7m-MoGM induced a T_H_1-biased response (online supplemental figure S5C). High-dose rlipoE7m-MoGM immunization also induced a higher frequency of IFN-γ-secreting cells than rlipoE7m immunization and increased the number of RAH-specific CD8^+^ T cells (online supplemental figure S5D, E). The s.c. rlipoE7m-MoGM treatment-induced tumor control is mediated by CD8^+^ T cells. Depletion of CD8^+^ cells in mice challenged with TC-1 tumors abrogated the antitumor effect induced by s.c. rlipo-E7m-MoGM therapy resulted in complete restoration of tumor outgrowth (figure 5C).

To further explore whether dual TLR2 agonist and GM-CSF stimulation effectively treated diverse syngeneic tumor models, we leveraged OVA as the tumor antigen to investigate TLR2 agonist and GM-CSF therapeutic effects on T cell thymoma (E.G7-OVA) and melanoma (B16F10-OVA), which represent aggressive and metastatic tumor models, respectively. We found that s.c. immunization with rlipo-OVA-MoGM therapy on day 7 post-tumor implantation successfully delayed E.G7 tumor outgrowth (figure 5D). To further assess the possibility that the combination of a TLR2 agonist and GM-CSF protects against metastatic cancer, we used a melanoma lung metastasis mouse model. Mice were s.c. immunized with recombinant proteins. Then, B16F10-OVA melanoma cells were intravenous injection to mice on day 7 postimmunization and analyzed melanoma lung metastasis on day 26 (post-tumor challenge day 19). Observation of tumor nodules in the lungs showed that rlipo-OVA-MoGM therapy provided significant protection against lung metastasis, as evidenced by the reduced number and size of tumor nodules compared with those in the control and rlipo-OVA-treated mice (figure 5E). Importantly, to mimic the therapeutic regimen, mice were given intravenous injection of B16F10-OVA melanoma cells on day 0, s.c. received recombinant protein therapy on day 3 and analyzed for melanoma lung metastasis on day 19. We also observed a reduction in tumor nodule formation in mice treated with rlipo-OVA-MoGM therapy compared with mice treated with rlipo-OVA (figure 5F). Hence, the combination of a TLR2 agonist and GM-CSF provided greater antitumor activity in established tumor models and protected against metastatic cancer.

### Systemic immunization with the TLR2 agonist and GM-CSF-containing fusion protein activates CD103^+^ DCs and reduces the number of immunosuppressive TAMs

**Figure 6:**
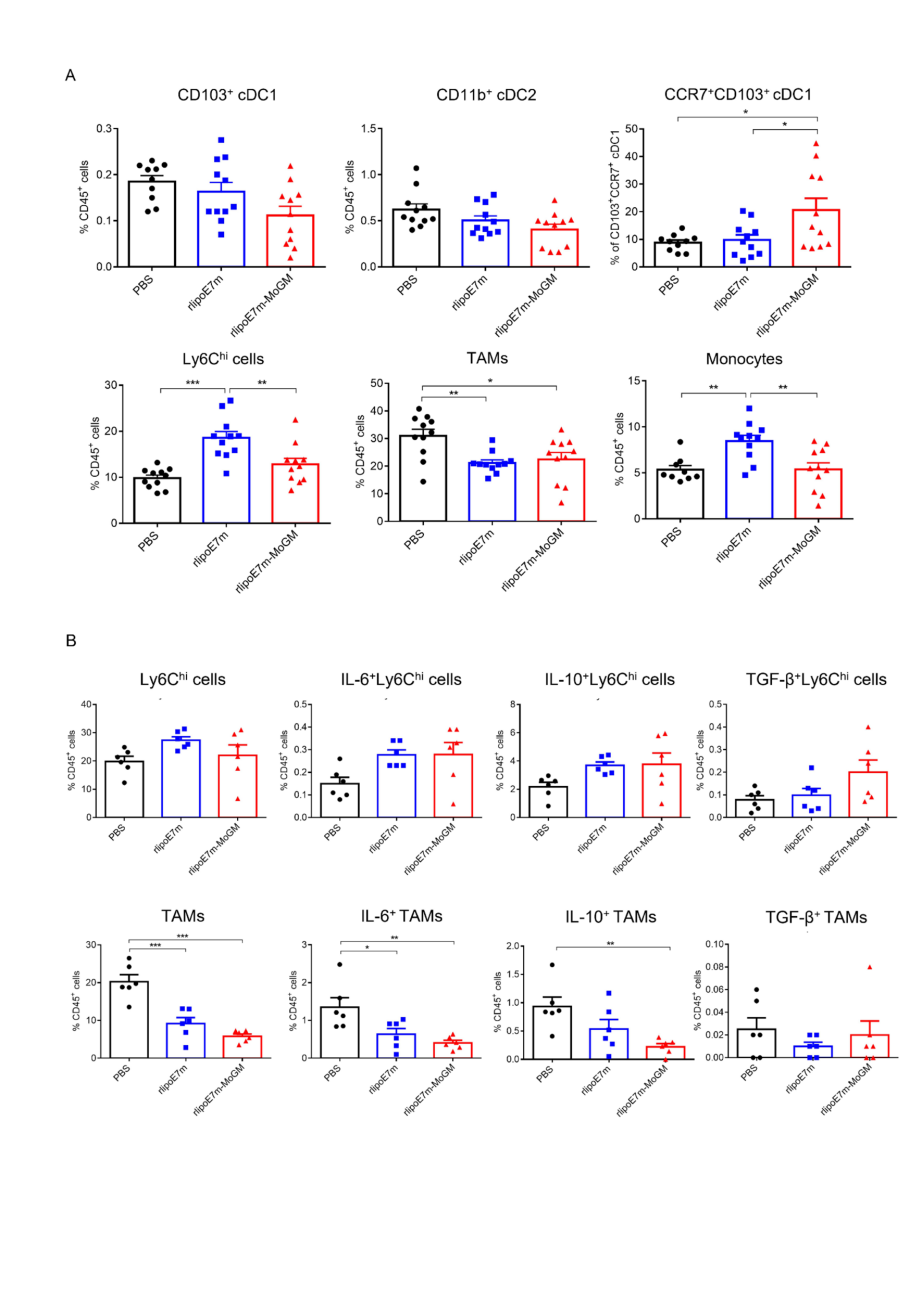
Effects of rlipoE7m-MoGM on tumor-infiltrating myeloid cells after s.c. immunization. TC-1 tumor-bearing mice were s.c. treated with the indicated recombinant proteins (1 nmol) on day 14 and sacrificed on day 22. (A) The tumor mass was digested for myeloid cell population analysis. The frequency of each myeloid cell subset within the CD45^+^ cell population in tumors was analyzed. (B) The single-cell suspension from tumor tissue was stimulated with LPS in the presence of brefeldin A for 8 hours, and intracellular cytokine staining was performed. The frequency of IL-6-, IL-10- and TGF-β-expressing myeloid cell subsets within the CD45^+^ cell population in tumors was analyzed. The data shown are presented as the mean±SEM of n=6 mice per group from a single independent experiment. *P<0.05, **p<0.01 and ***p<0.001 (one-way ANOVA with Tukey’s correction). ANOVA, analysis of variance; IL, interleukin; LPS, lipopolysaccharide; s.c., subcutaneous.

Consistent with the observation that a single systemic immunization induced regression of late-stage TC-1 tumors, we further explored how systemic rlipoE7m-MoGM administration affects tumor-infiltrating myeloid cells. To this end, tumors were isolated to assess the recruitment and activation of myeloid cell subsets 8 days after immunization. In the tumors, we observed that CCR7 expression was upregulated in CD103^+^ cDC1s after s.c. rlipoE7m-MoGM therapy, whereas CD103^+^ cDC1 and CD11b^+^ cDC2 subsets were not quantitatively affected by any of the systemic s.c. immunizations (figure 6A). In contrast, accumulation of Ly6c^hi^ cells and monocytes was observed in tumor lesions after rlipoE7m therapy. To further explore whether tumor-infiltrating myeloid cells secrete immunosuppressive cytokines due to the pleiotropic activity of GM-CSF, we collected a single cell suspension from tumor tissue and stimulated it with LPS in the presence of brefeldin A for 8 hours. We found no significant change in IL-6, IL-10 and TGF-β expression in Ly6c^hi^ cells. However, the percentages of IL-6^+^ TAMs and IL-10^+^ TAMs were reduced in rlipoE7m-MoGM-treated mice (figure 6B). These results suggest that the TLR2 agonist and GM-CSF synergistically reduced the number of immunosuppressive myeloid cells.

### Immunization with rlipoE7m-MoGM promotes tumor infiltration of antigen-specific T cells and enhances cytotoxic function

**Figure 7:**
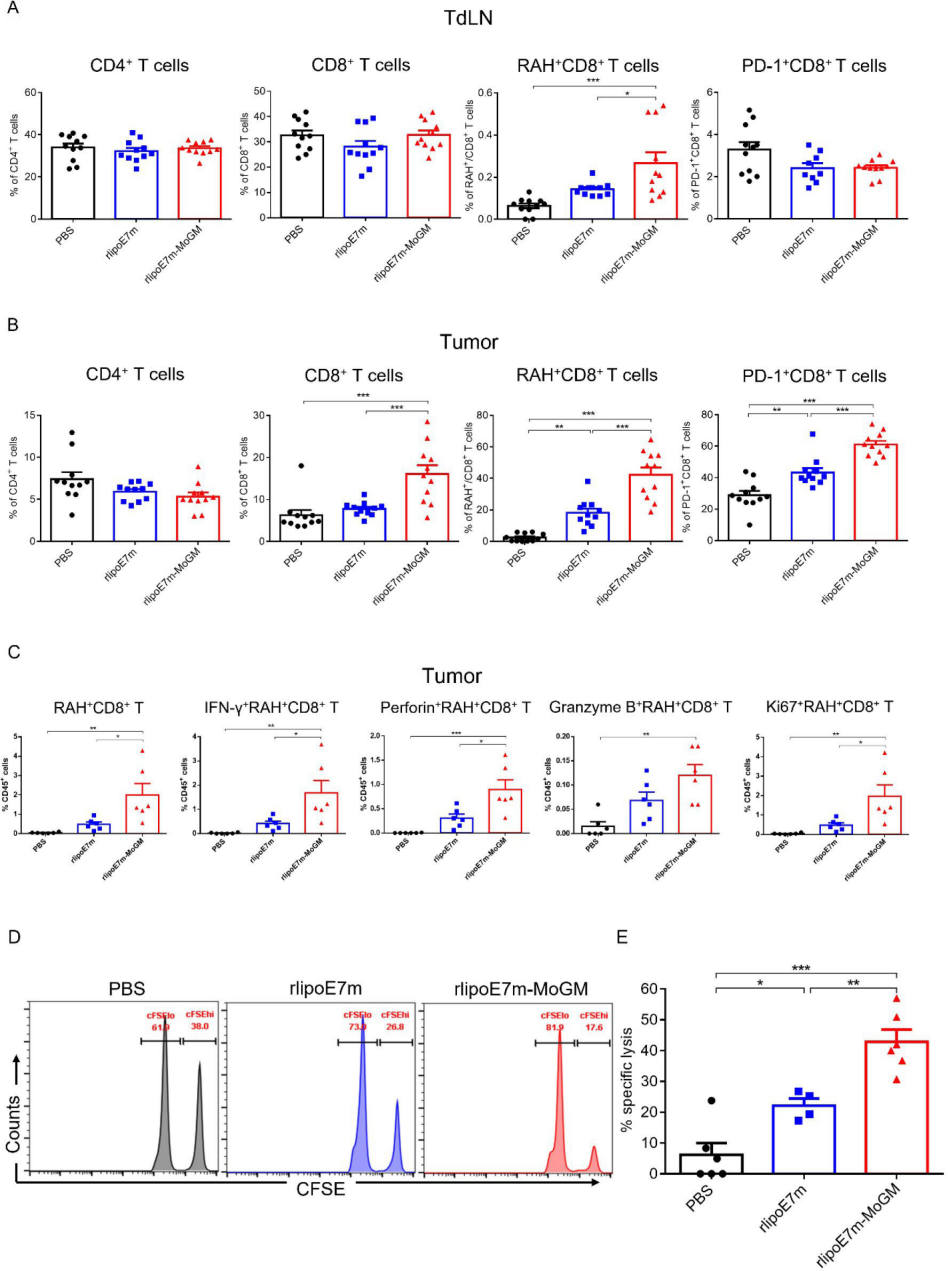
Dual TLR2 agonist and GM-CSF treatment promoted antigen-specific T cell proliferation and enhanced antigen-specific CTL responses. TC-1 tumor-bearing mice were s.c. immunization on day 14, and tumors were collected 8 days after immunization. (A, B) Measurements of infiltrating T lymphocytes by flow cytometry analysis in TdLNs (A) and tumors (B) were performed. (C) Single-cell suspensions from tumors were stimulated with RAH peptides, PMA and ionomycin in the presence of brefeldin A for 4–6 hours. IFN-γ, perforin, granzyme B and Ki67 expression in CD8^+^ T cells were determined by intracellular cytokine staining followed by flow cytometry analysis. (D, E) Vaccine-induced in vivo CTL killing assay. (D) Representative flow cytometry analysis of CFSE-labeled RAH^+^ target cells killed in the lymph nodes at 18 hours after adoptive transfer. (E) The percentage of target cells killed was determined. n=11–12 mice per group from two independent experiments in (A, B). *P<0.05, **p<0.01 and ***p<0.001 (one-way ANOVA with Tukey’s correction). ANOVA, analysis of variance; CFSE, carboxyfluorescein succinimidyl ester; CTL, cytotoxic T lymphocyte; GM-CSF, granulocyte/macrophage colony stimulating factor; RAH, RAHYNIVTF; TdLN, tumor-draining lymph nodes; TLR2, Toll-like receptor 2.

Next, we characterized whether activation of CD103^+^ cDC1s benefits rlipoE7m-MoGM therapy in terms of promoting CD8^+^ T cell infiltration into tumors. To this end, tumors and TdLNs were analyzed for the presence of RAH tetramer-specific CD8^+^ T cells by flow cytometry, and the gating strategy is shown in online supplemental figure S6. TC-1 tumor-bearing mice s.c. received either rlipoE7m or rlipoE7m-MoGM and showed no significant differences in the percentages of CD4^+^ T cells in either the TdLNs (figure 7A) or tumors (figure 7B). However, in tumor lesions, we detected high numbers of tumor-infiltrating CD8^+^ T cells from mice treated with rlipoE7m-MoGM therapy but not with rlipoE7m (figure 7B). Especially, rlipoE7m-MoGM therapy induced higher number of the RAH-specific CD8^+^ T cells with upregulated PD-1 expression in both the tumors and lymph nodes, showing antigen-specific CD8^+^ T cells were activated in response to rlipoE7m-MoGM treatment. To further assess the functional impact of rlipoE7m-MoGM treatment on CD8^+^ T cells, a single cell suspension from tumors was stimulated with RAH peptides, PMA and ionomycin in the presence of brefeldin A for 4–6 hours. We found that rlipoE7m-MoGM-treated mice, but not rlipoE7m-treated mice, showed an increased number of antigen-specific CD8^+^ T cells and highly expressed IFN-r and perforin within this population. Notably, antigen-specific CD8^+^ T cells proliferated in rlipoE7m-MoGM-treated tumors, as evidenced by Ki67 upregulation within this population. These results suggest that rlipoE7m-MoGM therapy exhibits enhanced cytotoxic CD8^+^ T cell function (figure 7C). To further evaluate the killing activity of vaccine-induced cytotoxic CD8^+^ T cells, we performed an in vivo CTL killing assay to quantitate the specific killing capacity of E7-specific cytotoxic CD8^+^ T cells by monitoring CFSE dilution. In this experiment, target cells were pulsed with a peptide or left unpulsed and then labeled CFSE^hi^ or CFSE^lo^. After adoptively transferring the target cells into mice 7 days after the second immunization, 42.9%±3.9% of the RAH-pulsed target cells were eliminated in rlipoE7m-MoGM-immunized mice compared with 22.2%±2.28% and 6.2%±3.8% of the target cells in rlipoE7m-immunized mice and control mice, respectively, indicating that rlipoE7m-MoGM enhances the function of antigen-specific CD8^+^ T cells and that most target cells were killed in rlipoE7m-MoGM-immunized mice (figure 7D, E). Importantly, these results demonstrate the dual functions of TLR2 agonist and GM-CSF-containing fusion protein immunization in enhancing the number and effector function of antigen-specific CD8^+^ T cells, which subsequently create an effective killing capacity.

10.1136/jitc-2021-002758.supp2 Supplementary data

## Discussion

Modulation of the immunosuppressive TME is critical to generate efficient tumor-specific immunity to control tumor growth. Here, we report that a TLR2 agonist and GM-CSF bifunctional adjuvant antigen can induce robust antitumor responses and reshape the immunosuppressive TME. The therapeutic use of TLR2 agonists and the immunostimulatory cytokine GM-CSF has been studied individually to assess antitumor efficacy. However, the crosstalk between the roles of a TLR2 agonist and GM-CSF in enhancing antigen-specific T cell responses is undefined. In this study, we found that GM-CSF and TLR2 agonist activities could synergistically enhance DC activation and lead to augmented CTL expansion, effector functions and antitumor efficacy. This improved therapeutic efficacy was dramatically increased on concurrent activation of CD103^+^ DCs and a decrease in the number of TAMs at the tumor site.

Our findings demonstrated that cooperative BMDC activation induced by the TLR2 agonist and GM-CSF upregulated the production of proinflammatory cytokines, including IL-6 and IL-1β. These results are consistent with those of our previous study showing that the TLR2 agonist rlipo-protein stimulates BMDCs to develop a proinflammatory phenotype.^5^ The TLR2 agonist in combination with GM-CSF further augments rlipo-protein-induced BMDC activation, which is consistent with previous reports showing that the proinflammatory properties of GM-CSF stimulate myeloid cells to increase production of IL-1β, IL-6 and TNF-α.^16^ However, inflammatory responses can either be beneficial for tumor immune control or promote tumor growth. Earlier studies established the protumor effect of TLR2 agonists that impairs antitumor immunity via IL-6-induced DC tolerance.^17^ It has been postulated that tumor progression is associated with chronic inflammation through a proinflammatory cytokine network.^18 19^ However, local administration of recombinant proteins enables acute and transient induction through TLR2 ligands, and the GM-CSF response can tune the antitumor response.^20^ Additionally, rlipoE7m-MoGM induced relatively high levels of IL-1β in BMDCs, ultimately potentiating a T_H_-1-type immune response, which suggests that the immunostimulatory benefits of combining TLR2 agonists and GM-CSF conferred to BMDCs outweigh the proinflammatory cytokine events related to tumorigenesis. The associations of these cytokines in tumor progression are context dependent.

Low numbers or dysfunction of CD8^+^ T cells in tumors counteract the efficacy of vaccine-induced antigen-specific T cell responses. In our study, the addition of GM-CSF considerably enhanced the TLR2 agonist-induced antitumor response by promoting the infiltration of antigen-specific cytotoxic T cells in the tumor and the amount of perforin and IFN-γ secreted. Importantly, the cooperative antitumor effect of TLR2 agonists and GM-CSF induces distant tumor regression, suggesting that local immunity can induce systemic regression of metastatic tumors. Furthermore, the induction of antitumor effects could represent immune memory in tumor-free mice (online supplemental figure S2). In addition, GM-CSF improved the T cell response, which has been well documented.^21 22^ Consistently, a study by Lee and colleagues reported that combined local GM-CSF administration and intramuscular E7 DNA treatment activated E7-specific CD8^+^ T cells, which accumulated in cervicovaginal TC-1 tumors in mice.^23^ The mode of action of GM-CSF in improving antitumor efficacy may be indirectly mediated through DC effects on both the priming and effector phases of T cell responses. Moreover, GM-CSF plays a role in T cell motility by regulating chemokine expression. A previous study demonstrated that GM-CSF upregulated CXCR3 expression on vaccine-induced T cells in humans.^24^ Additionally, the release of CXCL9/10, a ligand for CXCR3, was increased following TLR2 agonists plus IFN-γ stimulation in vitro.^25^ Further in vivo studies revealed that the combination of a TLR2 agonist and GM-CSF induced chemokine expression, which supports this possibility.

Multiple lines of evidence have established that tumor-infiltrating immune cells determine the prognosis of patients with cancer and the efficacy of therapy-induced immune responses. Analysis of cellular populations outlines the biological effects of a TLR2 agonist and GM-CSF in the TME. Of note, activation of tumor DCs, especially CD103^+^ cDC1s, is critically important for initiating the tumor-infiltrating CTL responses by cross-presenting acquired tumor antigens.^26–28^ Nonetheless, DCs represent a rare tumor population because tissue-resident CD103^+^ DCs fail to accumulate in injured tissue.^29^ In our study, rlipoE7m-MoGM therapy induced a broad immune response involving multiple myeloid cell types, such as DCs and macrophages. Consistent with reports showing that GM-CSF upregulates CCR7 chemokine receptor expression on DCs,^30^ we demonstrated that tumor CD103^+^ DCs from mice administered rlipoE7m-MoGM exhibited a high expression level of CCR7, suggesting that this treatment might provide critical tumor-specific T cell priming by transporting tumor antigens to the TdLNs.^31^ However, unlike the events seen with Flt3 ligand promoting the differentiation and expansion of CD103^+^ cDC1s,^32^ the number of CD103^+^ DCs was not quantitatively changed in the tumors following rlipoE7m-MoGM vaccination. A previous study showed that the development of DCs was determined by the GM-CSF quantity.^33 34^ Here, the low dose of GM-CSF might not be sufficient to promote CD103^+^ cDC1 development or recruit DCs without specific chemokine overexpression in tumors. Moreover, without DC activation and maturation through TLR signaling, a robust antitumor response may not be induced despite high tumor infiltration by DCs.^35^ Interestingly, treatment with rlipoE7m-MoGM reduced IL-6/IL-10-secreted TAMs but not Ly6C^hi^ myeloid cells. Because GM-CSF has been considered to shift macrophages into an M1-like phenotype,^36^ Ly6C^hi^ monocytes depend on M-CSF signaling for their recruitment and extravasation to tumors.^37^ These studies and our data suggest that the maturation status of i.t. DCs rather than the number of immature DCs infiltrating the tumor is a critical factor involved in triggering antigen-specific T cell responses.

Our findings highlight the role of macrophages in mediating antitumor efficacy in response to rlipoE7m-MoGM therapy. With local rlipoE7m-MoGM administration, a decreased frequency of TAMs in tumors was observed. This depletion of macrophages inhibited tumor growth and might result from the removal of a certain portion of protumor macrophages.^38^ GM-CSF expression reduces the responsiveness of macrophages to M-CSF by enhancing the cleavage of CSF-1R, preventing macrophage recruitment and proliferation.^30 39^ However, whether the combination of the TLR2 agonist and GM-CSF is involved in the function of macrophages, which can switch from protumor to antitumor phenotypes, needs to be further investigated.

## Conclusion

The combination of a TLR2 agonist and GM-CSF exhibits cooperative effects on DC activation and modulates TAM populations in the TME, which subsequently promote antigen-specific T cell infiltration in the tumor. On the basis of the encouraging efficacy and specificity shown, our study suggests that the combination of TLR2 agonists and GM-CSF is an excellent combinatorial adjuvant for triggering antitumor immunity and provides preclinical evidence of established tumor regression.

## Data Availability

Data are available on reasonable request. Data will be available from Dr. Shih-Jen Liu upon reasonable request.
