# Supplementary material for: Activation of GM-CSF and TLR2 signaling synergistically enhances antigen-specific antitumor immunity and modulates the tumor microenvironment
Source: J Immunother Cancer. 2021 Oct 1;9(10):e002758. doi: 10.1136/jitc-2021-002758 (PMC8488721; doi:10.1136/jitc-2021-002758)

Supplemental Figure Legends

Supplemental Figure S1 *In vitro* Characterization of purified rlipo-fused GM-CSF protein with tumor antigen.

(A) NFS-60 cells were incubated with serially diluted recombinant protein solution as indicated for 16 hours. Then, 1 μCi 3H-thymidine was added and incubated overnight to measure cell proliferation. The dose-response curve was calculated using the following equation: Y=Bottom + (Top-Bottom)/(1+10^((LogEC50-X)*HillSlope) using Prism 6. The data are represented as the mean ± SD of triplicate samples.

Supplemental Figure S2 Memory immunity on cured mice recovery from i.t. rlipoE7m-MoGM therapy and rechallenged with TC-1 tumor cells.

(A) The immunization protocol for TC-1 tumor cells rechallenged on cured mice. (B) Tumor growth of TC-1 tumor-bearing mice received i.t. rlipoE7m-MoGM therapy. (C) Tumor growth of TC-1 tumor cells rechallenged on the contralateral flank of cured mice recovery from i.t. rlipoE7m-MoGM therapy. The naïve mice were as a control for tumor rechallenge. The data is from a single experiment and presented as the mean ± SEM. *P<0.05, **P<0.01 and ***P<0.001 [2-way ANOVA with Tukey's correction].

Supplemental Figure S3 Design, production, characterization of rlipo-OVA-MoGM.

(A) Schematic diagram of rlipo-OVA-MoGM. The OVA gene was cloned into the pET22b vector along with the lipidation signal peptide at the N-terminus and a hexahistidine tag (HisTag) at the C-terminus with mouse GM-CSF. (B) The recombinant fusion protein was expressed in *E. coli* and purified with Ni-NTA. The protein purity in each step was analyzed by 10% SDS-PAGE (left) and detected with anti-His Tag antibodies (right). I: IPTG induction; NI: Noninduction; Extraction: 6 M guanidine HCl (GuHCl) extraction; Elution: 500 mM imidazole elution. (C) The N-terminal lipid moiety of rlipo-OVA-MoGM was analyzed on a MALDI micro MX^{TM} mass spectrometer. The MALDI-TOF MS spectra present three lipid peptide signal peaks: 1452, 1466, and 1480 *m/z*.

Supplemental Figure S4 Myeloid cell populations in TdLNs after i.t. treatment with rlipoE7m-MoGM.

TC-1 tumor-bearing mice were treated with the indicated recombinant proteins on days 14, 16 and 18. The mice were sacrificed on day 20, and the TdLNs were digested for cell population analysis. The frequency of each myeloid cell subset within the

CD11c$^+$MHCII$^+$ cell population in TdLNs was analyzed. Data are shown as the mean ± SEM of n=4-6 mice per group from 2 independent experiments.

Supplemental Figure S5 The potency of rlipoE7m-MoGM vaccination-induced antigen-specific CTL responses.

Naïve C57BL/6 mice were s.c. immunization with 1 nmol (A and B) or 4 nmol (C and D) recombinant proteins twice at weekly intervals. Seven days after the final immunization, the spleens from immunized mice were collected and restimulated with rE7m (10 μg/mL) for 5 days for ELISA. (A) The supernatants were collected for IFN-γ ELISA. (B) Splenocytes were restimulated for 48 hours with RAH peptides (D^b-RAHYNIVTF, 10 μg/ml) or irrelevant peptides in an anti-IFN-γ-coated ELISpot plate. (C) The supernatants of splenocytes from 4 nmol recombinant protein immunization were assayed for IFN-γ and IL-5 ELISA. (D) Splenocytes from 4 nmol recombinant protein immunization were assayed for IFN-γ using ELISpot. (E) Cells were stained with PE-conjugated RAH tetramer, anti-CD8-BV510 and anti-CD19-APC antibodies. The percentage of RAH-specific CD8$^+$ T cells was calculated by flow cytometry with the exclusion of CD19$^+$ cells. The data were compiled from two independent experiments and are presented as the mean ± SEM. *P<0.05, **P<0.01 and ***P<0.001 [2-way ANOVA with Tukey's correction]

Supplemental Figure S6 Gating strategy of flow cytometry for intracellular staining of tumor-infiltrating T cells. (A) Gating strategy of tumor-infiltrating T cells. (B) The single-cell suspensions from tumors were ex vivo treated with RAH peptides, PMA and ionomycin in the present of brefeldin A for 4-6 h. **.**The percentages of tumor-infiltrating IFN-γ$^+$CD8$^+$ T cells, perforin$^+$CD8$^+$ T cells, granzyme B$^+$CD8$^+$ T cells and Ki67$^+$CD8$^+$ T cells within the CD45$^+$ population were analyzed. Representative flow cytometry graphs of CD8$^+$ T cells are shown.

Supplemental Figure S1

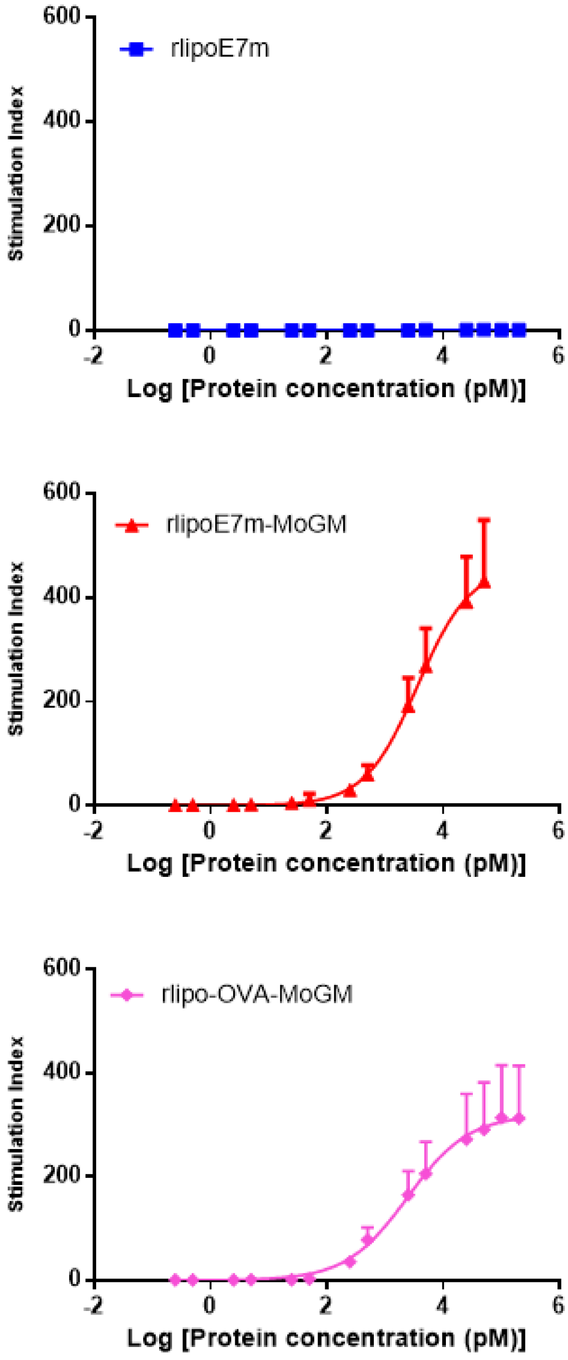

Supplemental figure S2

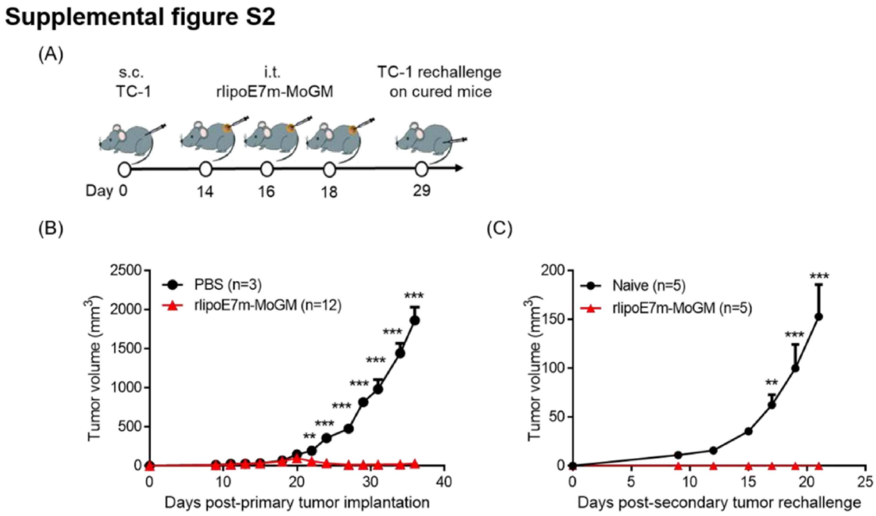

Supplemental Figure S3

(A)

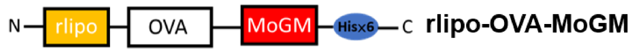

(B)

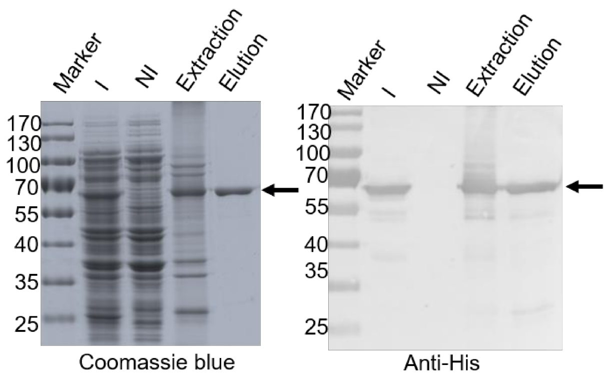

(C)

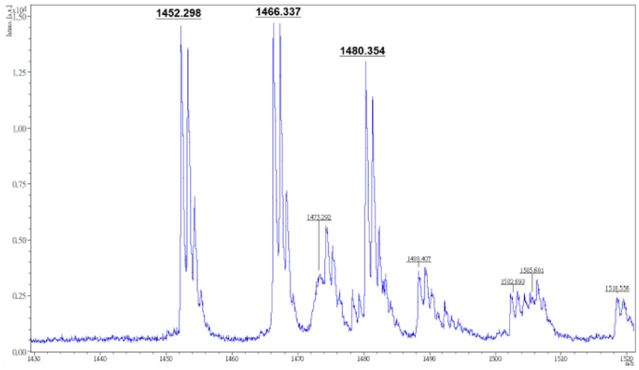

Supplemental figure S4

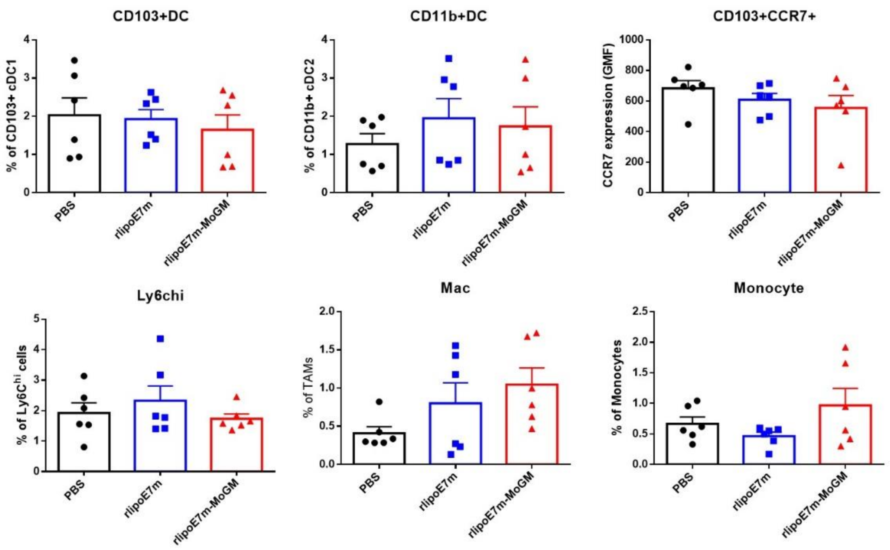

Supplemental Figure S5

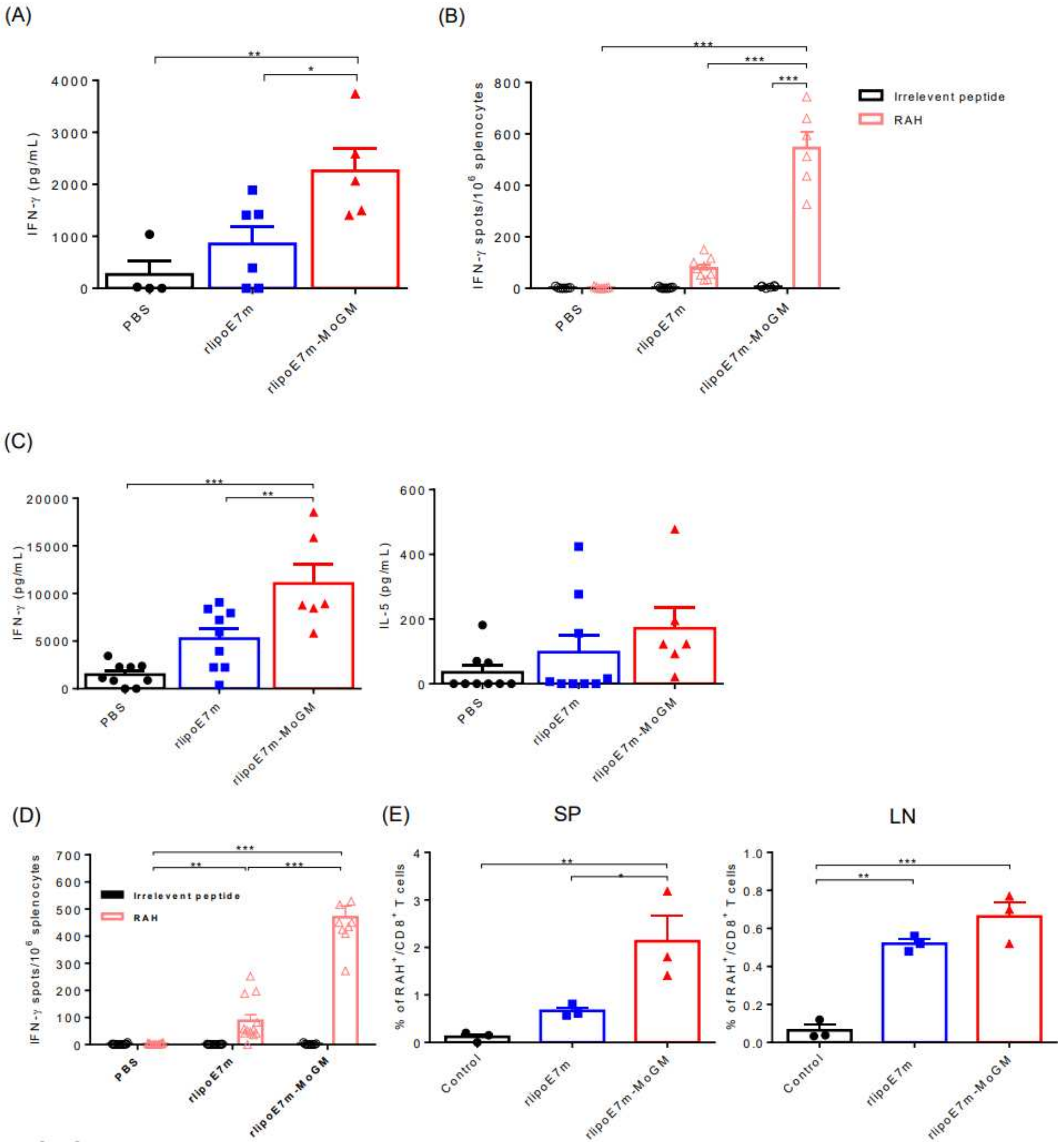

Supplemental Figure S6

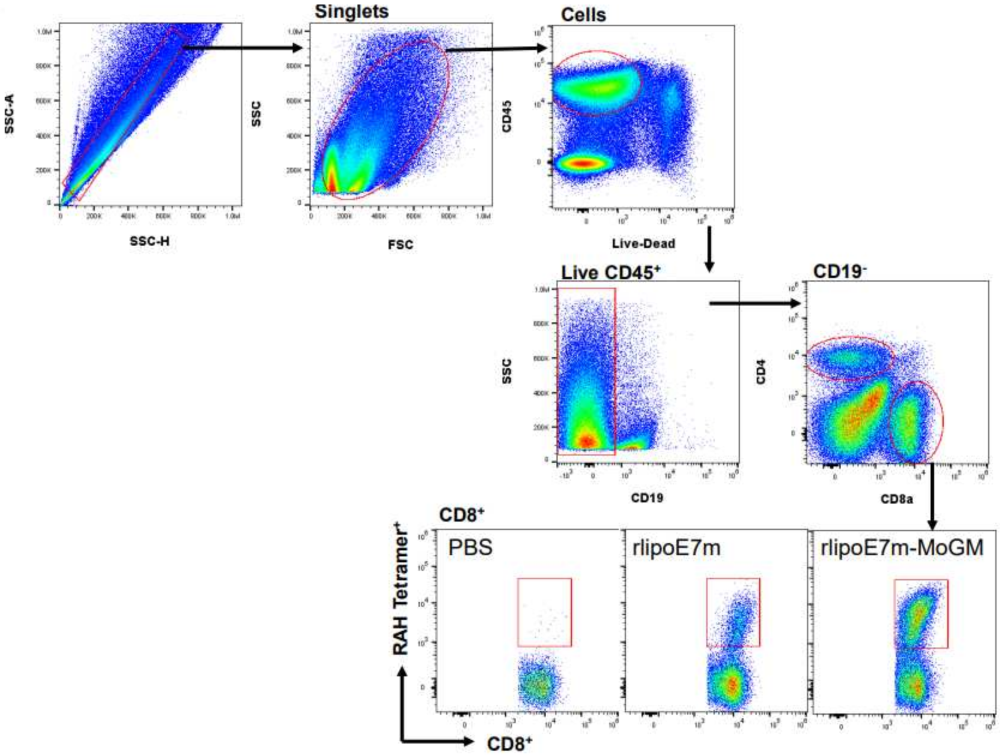

Supplement: Supplementary data [file jitc-2021-002758supp001.pdf]
